# Determinants of Arterial Stiffness in Chronic Kidney Disease Stage 3

**DOI:** 10.1371/journal.pone.0055444

**Published:** 2013-01-30

**Authors:** Natasha J. McIntyre, Richard J. Fluck, Christopher W. McIntyre, Apostolos Fakis, Maarten W. Taal

**Affiliations:** 1 Department of Renal Medicine, Royal Derby Hospital, Derbyshire, United Kingdom; 2 Department of Vascular Medicine, University of Nottingham, Nottingham, United Kingdom; Lausanne University Hospital and University of Lausanne, Switzerland

## Abstract

**Background:**

Early chronic kidney disease (CKD) is associated with increased cardiovascular (CV) risk but underlying mechanisms remain uncertain. Arterial stiffness (AS) is associated with increased CV risk in advanced CKD, but it is unclear whether AS is relevant to CV disease (CVD) in early CKD.

**Study Design:**

Cross-sectional.

**Setting and participants:**

1717 patients with previous estimated glomerular filtration rate (eGFR) 59–30 mL/min/1.73 m^2^; mean age 73±9y, were recruited from 32 general practices in primary care.

**Outcomes:**

Increased arterial stiffness.

**Measurements:**

Medical history was obtained and participants underwent clinical assessment, urine and serum biochemistry testing. Carotid to femoral pulse wave velocity (PWV) was determined as a measure of AS, using a Vicorder™ device.

**Results:**

Univariate analysis revealed significant correlations between PWV and risk factors for CVD including age (r = 0.456; p<0.001), mean arterial pressure (MAP) (r = 0.228; p<0.001), body mass index (r = −0.122; p<0.001), log urinary albumin to creatinine ratio (r = 0.124; p<0.001), Waist to Hip ratio (r = 0.124, p<0.001), eGFR (r = −0.074; p = 0.002), log high sensitivity c-reactive protein (r = 0.066; p = 0.006), HDL (r = −0.062; p = 0.01) and total cholesterol (r = −0.057; p = 0.02). PWV was higher in males (9.6 m/sec vs.10.3 m/sec; p<0.001), diabetics (9.8 m/sec vs. 10.3 m/sec; p<0.001), and those with previous CV events (CVE) (9.8 m/s vs. 10.3 m/sec; p<0.001). Multivariable analysis identified age, MAP and diabetes as strongest independent determinants of higher PWV (adjusted R^2^ = 0.29). An interactive term indicated that PWV increased to a greater extent with age in males versus females. Albuminuria was a weaker determinant of PWV and eGFR did not enter the model.

**Limitations:**

Data derived from one study visit, with absence of normal controls.

**Conclusion:**

In this cohort, age and traditional CV risk factors were the strongest determinants of AS. Albuminuria was a relatively weak determinant of AS and eGFR was not an independent determinant. Long-term follow-up will investigate AS as an independent risk factor for CVE in this cohort.

## Introduction

Multiple epidemiological studies attest that chronic kidney disease (CKD) is associated with increased cardiovascular risk compared to the general population, and may account for up to 50% of all deaths in this group [1]. In many studies people with early stage CKD are more likely to die from cardiovascular disease than progress to end stage kidney disease (ESKD) [2]. Hallan [3] reported that the risk of CKD progression is low until eGFR falls below 30 ml/min/1.73 m^2^. In contrast even modest reductions in eGFR are incrementally associated with reduced survival [4]. The increased cardiovascular (CV) risk associated with advanced stages of CKD cannot be explained by traditional risk factors alone, but is attributable to a combination of traditional and non-traditional factors [5], [6]. Arterial stiffness (AS) has been identified as one non-traditional risk factor associated with the large cardiovascular risk burden in CKD [7], [8]. Arterial stiffness in CKD is proposed to provoke an increase in systolic blood pressure (SBP) and pulse pressure (PP). This in turn leads to an increase in ventricular afterload, myocyte hypertrophy and reduced coronary perfusion, resulting in systolic and diastolic dysfunction. Elevated systolic and pulse pressures may also contribute to vascular damage, further increasing CV risk [9].

Aortic pulse wave velocity (aPWV) is a measure of AS and has predicted cardiovascular morbidity and mortality in a number of populations including the healthy elderly and people with hypertension, diabetes or ESKD on haemodialysis [10], [11], [12], [13], [14]. On the other hand, data regarding the relationship between AS and CKD in earlier stages appear conflicting. Several studies have reported an increase in arterial stiffness and CV risk associated with early CKD [15], [16], [17] but others have not [18], [19], [20]. More data are therefore required regarding the relationship between AS and markers of kidney disease in early stage CKD. The aim of our study was to investigate if previously identified determinants of AS are also relevant in a population of predominantly elderly people with CKD stage 3, representing the majority affected by CKD.

## Study Population and Methods

### Participants and Recruitment

We studied 1741 patients with CKD stage 3 recruited from general practitioner (GP) practices. The methods have previously been described in detail and are summarised here with emphasis on the measurement of aPWV [21], [22]. Participants were recruited as part of the Renal Risk in Derby (RRID) study, a prospective cohort study planned to continue for 10 years, with the aim of studying renal and CV risk factors in patients with CKD stage 3 in a primary care setting. Eligible participants were 18 years or over, met the KDOQI criteria for CKD stage 3 (eGFR of 30–59 mL/min/1.73 m^2^ on 2 or more occasions at least 3 months apart), were able to give informed consent and attend their GP practice for assessments. People who had previously received a solid organ transplant or who were terminally ill (expected survival <1 year) were excluded. The RRID study is being conducted by a single Nephrology Department, but participants were recruited directly from 32 GP practices in Derbyshire, UK. Study visits were conducted at participating GP practices by the researchers. Twenty four participants were unable to have their aPWV measured due to a combination of technical failure or inability of the participant to lie at a 30 degree angle. Thus 1717 participants with aPWV readings were included in this analysis.

### Data Collection

First study visits were conducted from August 2008 to March 2010. Screening and baseline visits were combined due to the large proportion of elderly participants and the logistical challenges associated with conducting study visits in multiple primary care centres. Participants were sent a medical questionnaire as well as 3 urine specimen bottles, and were asked not to eat cooked meat for at least 12 hours prior to the assessment [23]. At the assessment information on questionnaires was checked, anthropomorphic measurements and blood pressure taken, and urine analysis was performed. Blood and urine specimens were submitted for biochemical analysis to a single laboratory.

Previous diabetes was defined by having a previous clinical diagnosis according to WHO criteria [24]. Previous cardiovascular event (CVE) was defined as subject reported myocardial infarction, stroke, transient ischaemic attack, revascularisation or amputation due to peripheral vascular disease, or aortic aneurysm. Anaemia was defined as hemoglobin <11.5 g/dL for women and <13.5 g/dL for men. Obesity was defined as BMI>30 kg/m^2^. Central fat distribution was defined as a waist to hip ratio (WHR) of ≥0.9 for men or ≥0.8 for women [25].

#### Blood pressure

Blood pressure (BP) was measured after a minimum of five minutes rest in the sitting position, using a calibrated oscillometric device, recommended by the British Hypertension Society (Digital Blood Pressure Monitor Model UA-767, A&D Instruments Ltd, Abingdon, UK). A single device was used for all readings. Measurements were taken until three readings that were within 10% of each other were obtained. BP was calculated as the mean of these three readings. Hypertension was defined as a systolic BP≥140 mmHg, diastolic BP≥90 mmHg, or current antihypertensive medication [26]. Mean arterial pressure (MAP) was calculated as 1/3 the average SBP plus 2/3 the average DBP.

#### Pulse wave velocity

Carotid to femoral pulse wave velocity was measured as a marker of arterial stiffness, a critical determinant of cardiovascular outcomes in CKD [7], [27], [28], and considered the gold standard measurement of AS [12]. Measurements were performed using a Vicorder™ device (Skidmore Medical Ltd, Bristol, UK). The Vicorder™ is small, portable, non-invasive and non-operator dependant making it well suited for use in community based studies. Readings take only 2–3 minutes to complete. Assessments were performed after at least 5 minutes of rest, according to manufacturers’ instructions in the semi-prone position (at approximately 30°) to prevent venous contamination of the arterial signal. The participant had a neck-pad placed around their neck with the pressure pad over the right carotid area. A blood pressure cuff was placed around the patients’ upper right thigh. The distance between the supra-sternal notch and the thigh cuff was measured using the direct method. To eliminate the potential effect of abdominal obesity on the distance measurement, an imaginary line was drawn from the supra-sternal notch to the right shoulder and the measurement to the thigh cuff was made along the side of the body. The neck-pad and thigh cuff were inflated by the Vicorder to 60 mmHg and then deflated to obtain a pressure tracing. Aortic PWV is calculated by the Vicorder by comparing carotid and femoral pressure tracings after a stable pattern is obtained. The mild discomfort caused by the inflation of a cuff placed around the neck precluded us from doing multiple readings. The intra-observer coefficient of variation for PWV measurements was 6.3%.

### Skin Autofluorescence

Skin autofluorescence (AF), a measure of skin Advanced Glycation Endproduct (AGE) deposition that has been identified as a marker of cumulative metabolic stress [29], [30], [31], was assessed on the left forearm using an AGE Reader™ device (DiagnOptics, Groningen, Netherlands). Three readings were taken and the average calculated. It was not possible to conduct these readings on dark skin. Values are expressed in arbitrary units [22].

#### Albuminuria

Albuminuria was assessed by measuring the urine albumin to creatinine ratio (ACR) on three consecutive early morning urine specimens collected prior to the clinic visit and stored in a refrigerator. Microalbuminuria was defined as a mean urine ACR of >2.5 mg/mmol in males or >3.5 mg/mmol in females and overt albuminuria as >30 mg/mmol [23].

#### Estimation of glomerular filtration rate (eGFR)

Biochemical assessments were performed by autoanalyser in a single laboratory. The creatinine assay has been standardised against an isotope dilution mass spectrometry (IDMS) method and the 4-variable MDRD equation modified for use with IDMS standardised creatinine measurement was used to estimate GFR. This study was commenced prior to publication of the CKD-EPI formula and eGFR derived from the MDRD formula was therefore used for recruitment and baseline study assessments.

The study was approved by the Nottingham Research Ethics Committee 1 and abided by the principles of the Declaration of Helsinki. All participants provided written informed consent. The study was included on the National Institute for Health Research (NIHR) Clinical Research Portfolio (NIHR Study ID:6632) and was independently audited by QED Clinical Services in November 2009.

### Statistical Analysis

Results presented are a cross sectional analysis of data from the first study visit. Continuous variables are reported as the mean and standard deviation (SD) if normally distributed or the median and inter-quartile range (IQR) if not. A t-test was used to compare two groups where variables were normally distributed and a Mann U Whitney test used if not. Pearson’s test was used to assess univariate correlations with aPWV for variables with normal distribution or Spearman’s test if distribution was not normal. Partial correlations were used to correct for MAP. Variables with skewed distribution (exponential) were log transformed for the correlations. SPSS version 15.0 was used for univariate analysis. P<0.05 was considered statistically significant.

Multivariable linear regression analysis, using the forward stepwise method, was used to identify independent determinants of arterial stiffness. P<0.05 was used for a variable to enter the model. The model was built using a hierarchical approach that introduced variables one-by-one on the basis of biological plausibility as well as strength of association in univariate analyses adjusted for age and diabetes. Nested models were tested using the Akaike Information Criterion (AIC). Models with a smaller AIC value were selected. An interactive term for age by gender was also tested in the final model. A scatter plot of regression residuals versus predicted values and Cook’s Distance plot were used for testing the assumptions of linear regression and identifying any outliers. The adjusted R-squared value is reported as a measure of goodness-of-fit. The regression coefficients (95% Confidence Intervals) and standardised coefficients (Beta) from the final multivariable model are presented. STATA version 11.1 was used for multivariable analysis.

## Results

Baseline characteristics are summarised in Table 1. Over a third of participants were obese (37%). Most (88%) had hypertension and 65% were treated with a renin angiotensin aldosterone system inhibitor (RAASi).

**Table 1:** Baseline characteristics.

	Females	n	Males	n	Total	n
Age (years)	73(66–79)	1037	75(69–80)*	680	74(67–79)	1717
Ethnicity white	1014 (98)	1037	661 (97)	680	1675 (98)	1717
Diabetes	158 (15)	1037	129 (19)*	680	287 (17)	1717
Previous CVE	185 (18)	1037	195 (29)*	680	380 (22)	1717
Current smoker	47 (5)	1037	31 (5)	680	78 (4.5)	1717
Previous smoker	407 (39)	1037	444 (65)*	680	851 (50)	1717
BMI (kg/m^2^)	29.0(25.6–32.5)	1037	27.8(25.7–30.5)*	680	28.4(25.6–31.8)	1717
Systolic BP (mmHg)	133±19	1037	136±18*	680	134±18	1717
Diastolic BP (mmHg)	72±11	1037	74±11*	680	73±11	1717
MAP (mmHg)	92±12	1037	94±11*	680	93±12	1717
eGFR (mL/min/1.73 m^2^)	54.4(46.7–61.1)	1037	51.1(44.1–57.6)*	680	53.3(45.5–59.8)	1717
HDL Cholesterol (mmol/L)	1.55(1.27–1.85)	1032	1.19(1.00–1.45)*	676	1.40(1.13–1.71)	1708
Total Cholesterol (mmol/L)	4.90(4.10–5.80)	1032	4.20(3.60–4.98)*	676	4.6(3.9–5.5)	1708
Serum total protein (g/L)	74.2±4.8	1031	74.4±4.9	677	74.3±4.9	1708
Serum albumin (g/L)	40.5±3.0	1036	40.9±3.3*	678	40.7±3.2	1714
Corrected calcium (mmol/L)	2.39±0.10	1033	2.35±0.09*	673	2.38±0.10	1706
Phosphate (mmol/L)	1.15±0.17	1018	1.05±0.18*	667	1.11±0.18	1685
UACR (mg/mmol)	0.23(0.00–0.90)	1035	0.63(0.00–3.27)*	679	0.33(0.00–1.50)	1714
CKD 3B	208 (20)	1037	194 (29)*	680	402 (23)	1717
aPWV (m/sec)	9.7±2.0	1037	10.3±2*	680	9.9±2	1717
hs CRP (mg/L)	2.23(1.13–4.67)	1036	2.21(1.14–4.28)	680	2.22(1.13–4.50)	1716
Waist to Hip Ratio	0.86(0.81–0.90)	1037	0.98(0.94–1.02)*	680	0.91(0.84–0.97)	1717
SAF (AU)	2.60(2.30–3.00)	1023	2.73(2.33–3.18)*	665	2.67(2.30–3.07)	1688

Data are mean±SD, median (IQR) or number (%).

* P<0.05 versus females.

aPWV = aortic pulse wave velocity; AU = Arbitrary units.; BP = blood pressure; BMI = body mass index; CKD 3B = eGFR 30–44 mLs/min/1.73 m^2^; CVE = cardiovascular event; eGFR = estimated glomerular filtration rate; HDL = high density lipo-protein; hs CRP = high sensitivity C-reactive protein; MAP = mean arterial pressure; SAF = skin autofluorescence; UACR = urine albumin to creatinine ratio.

Mean aPWV was 9.9±2 m/sec. Aortic PWV was significantly higher in participants who were male, had diabetes, had had previous CVE, had albuminuria (microscopic or overt), were hypertensive, were not obese (as defined by BMI), had a blood pressure over 130/80 mmHg or were ≥75 years of age (Table 2). People who evidenced increased central fat distribution (central obesity), anaemia, were receiving antihypertensive medication, had CKD stage 3B or who had previously smoked also had significantly higher aPWV (Table 2). Age evidenced the strongest correlation with aPWV before and after adjusting for MAP (Table 3). Mean arterial pressure was also strongly correlated with aPWV. Other significant correlations are shown in Table 3. Adjustment for MAP produced little change in the correlations.

**Table 2:** aPWV readings in selected subgroups.

	Yes	No	P value
Male	10.3±2.0	9.7±2.0	<0.001
Diabetes	10.3±2.0	9.8±2.0	<0.001
Previous CVE	10.3±0.7	9.8±0.6	<0.001
Albuminuria*	10.3±2.0	9.8±2.0	<0.001
Hypertension‡	10.0±2.0	9.1±2.0	<0.001
BP<130/80 at baseline visit	9.2±1.7	10.3±2.1	<0.001
Obese	9.5±1.9	10.1±2.0	<0.001
Age <75 years	9.1(8.1–10.3)	10.5(9.4–11.8)	<0.001
Anaemia	10.2±1.9	9.8±2	0.003
Central Obesity	9.9±2	9.5±1.9	0.003
On antihypertensive medication	10.0±2.0	9.6±2.1	0.007
CKD stage 3B	10.1±2	9.8±2.1	0.009
Previous Smoker	10.0±2.1	9.8±1.9	0.011

Data are presented as mean ± standard deviation or median (interquartile range).

aPWV (aortic pulse wave velocity) readings expressed as m/sec.

* Microalbuminuria or greater levels of proteinuria.

CKD 3B = eGFR 30–44 mls/min/1.73 m^2^; CVE = cardiovascular event;

MAP = Mean arterial pressure;

‡ = Hypertension at entry into study;

Mann U Whitney tests used where data were not normally distributed.

**Table 3:** Significant Correlations with aPWV.

	Total Cohort(unadjusted)		Total Cohort(adjusted for MAP)	
	r	P-value	r	P-value
Age (years)	0.456*	<0.001	0.461	<0.001
MAP (mmHg)	0.228	<0.001	–	–
Urine ACR (mg/mmol)‡	0.124	<0.001	0.111	<0.001
Waist to Hip ratio‡	0.124	<0.001	0.121	<0.001
BMI (kg/m^2^)‡	−0.122	<0.001	−0.133	<0.001
Skin autofluorescence‡	0.117	<0.001	0.133	<0.001
Total Protein (g/L)	0.084	0.001	0.049	0.046
eGFR (mL/min/1.73 m^2^)	−0.074*	0.002	−0.096	<0.001
hs CRP‡	0.066	0.006	0.057	0.020
HDL Cholesterol (mmol/L)‡	−0.062	0.010	−0.069	0.005
Total Cholesterol (mmol/L)‡	−0.057	0.018	−0.100	<0.001

* Spearman correlation, otherwise Pearson correlation used. For adjusted values partial correlations were used.

‡ = log transformed data. r = correlation coefficient.

ACR = albumin to creatinine ratio; aPWV = aortic pulse wave velocity; BP = blood pressure; BMI = Body Mass Index; eGFR = estimated glomerular filtration rate; IMD = Indicies of multiple deprivation; HDL = high density lipo-protein; HS CRP = High sensitivity C-reactive protein; MAP = Mean arterial pressure.

Multivariable analysis was performed to identify independent determinants of a higher aPWV (Table 4; adjusted R^2^ = 0.29). The strongest and most significant of these were age, MAP, diabetes and BMI. Interestingly, BMI showed a negative relationship with aPWV. Other relatively weak independent determinants included serum total protein, log urine ACR and HDL cholesterol. An interactive term indicated that PWV increased to a greater extent with age in males versus females.

**Table 4:** Independent Determinants of higher aPWV.

	Total CohortAdjusted R^2^ = 0.29		
	B (95% CI)	β	p value
Age (10 years)	1.09 (0.94 to 1.24)	0.49	<0.001
Gender (female)	1.46 (0.07 to 2.85)	0.35	0.04
MAP (10 mmHg)	0.43 (0.35 to 0.50)	0.24	<0.001
Diabetes Mellitus	0.61 (0.38 to 0.83)	0.11	<0.001
Body Mass Index (kg/m^2^)	−0.042 (−0.058 to −0.025)	−0.11	<0.001
Total serum protein	0.025 (0.0084 to 0.042)	0.06	0.003
‡UACR (mg/mmol)	0.060 (0.0061 to 0.11 )	0.05	0.03
HDL Cholesterol (mmol/L)	−0.21 (−0.42 to −0.0028)	−0.046	0.047
Interactive term age bygender (F)	−0.22 (−0.41 to −0.031)	−0.39	0.02

B = un-standardised coefficient (95% confidence intervals).

β = standardized coefficient (Beta).

F = female.

‡ log-transformed data.

aPWV = aortic pulse wave velocity; HDL = high density lipo-protein; MAP = mean arterial blood pressure; UACR = urine albumin to creatinine ratio.

Independent variables that did not enter the equation were: eGFR, previous smoker status, past history of cardiovascular event, anaemia, white cell count, serum potassium, total cholesterol, skin autofluorescence, log urine protein to creatinine ratio, log high sensitivity CRP, waist to hip ratio, serum glucose. treatment with inhibitors on the renin angiotensin aldosterone system (RAASi), antihypertensive treatment.

## Discussion

We have identified age as the strongest determinant of arterial stiffness in this cohort of predominantly elderly patients with CKD stage 3. Other significant independent determinants of aPWV included traditional cardiovascular risk factors such as blood pressure, diabetes, obesity and a marker of dyslipidaemia. Markers of CKD were associated with aPWV only in univariate analysis (eGFR) or were weak determinants of aPWV (albuminuria). Our data therefore suggest that markers of kidney disease are not strong determinants of AS in early CKD and that traditional risk factors for CVD may be more important, or that mechanisms unrelated to AS mediate the association between early CKD and increased cardiovascular risk in this population.

Published data regarding the relationship between AS and CKD appear contradictory. Studies of patients receiving dialysis or with advanced CKD reported significantly increased AS compared with the general population [32], [33] but results from studies that included those with earlier stages of CKD are variable. A number of studies have reported associations with reduced GFR and increased AS [15], [17], [34]. In a relatively small study of 102 people with a wide spectrum of CKD (stages 1–5) a clear stepwise increase corresponding to stage of CKD was reported [15]. Multivariable analysis confirmed an independent association between GFR and aPWV; however only a small number of participants (n = 45) had CKD stage 3–4 and GFR was substantially lower than in our cohort (mean eGFR of 38 mL/min/1.73 m^2^). In a larger study of 2564 patients with CKD who were not receiving dialysis (almost half with diabetes, mean age 60.7 years, mean eGFR 40.7 mL/min/1.73 m^2^), Townsend et al. also found an increase in aPWV with declining GFR [17]. Similarly, a population-based study of 767 people (mean age 68 years, eGFR 60.6 mL/min/1.73 m^2^ and ACR 0.57 mg/mmol) targeting screening for type 2 diabetes, found that AS increased as GFR decreased in those with mild CKD (stage 2–3) [34]. In addition urinary ACR was positively associated with increased AS. On the other hand Briet et al [18] reported that AS was higher in patients with CKD than in hypertensive patients without CKD, but in the analysis of patients with CKD stage 3–5 they did not find a significant relationship between measured GFR and aPWV. Similarly, in a study of 150 patients with CKD stages 2–5D, aPWV was significantly higher in patients with CKD versus controls without CKD, but aPWV was not higher in those with more advanced CKD [20]. In another study of patients with CKD, blood pressure was the major determinant of PWV and although PWV correlated with GFR in a univariate analysis, GFR was not an independent determinant of PWV in a multivariable analysis [35]. Similarly, in 113 patients with CKD, PWV increased with increasing number of components of the metabolic syndrome irrespective of GFR [36]. In one analysis of data from the Framingham Heart Study that included 181 patients with early CKD and characteristics very similar to ours (mean age 70 years, mean eGFR 51 mL/min/1.73 m^2^, median urinary ACR 10 mg/g), AS was not different between those with or without CKD (defined by reduced GFR) after multivariable adjustment at baseline. In a longitudinal analysis, increased AS was not associated with increased risk of developing CKD [6]. On the other hand, higher aPWV was associated with elevated urinary albumin excretion at baseline and increased risk of developing microalbuminuria. Finally, in the Nephro Test cohort of 180 patients with CKD (mean age 59.6years, eGFR 32 mL/min/1.73 m^2^) aortic PWV remained stable during 3.5 years of follow up despite a significant decline in GFR and an increase in albuminuria. Interestingly, increased carotid circumferential wall stress and pulse pressure were associated with a greater risk of progression to ESKD [19]. Taken together, published data show that arterial stiffness increases in advanced stages of CKD but that changes are more variable in early stages, probably reflecting differences in the populations studied, particularly with respect to age. Thus the lack of an independent negative association between eGFR and increased aPWV in our study as well as the weak association between urinary ACR and increased aPWV are probably attributable to the fact that our study population was predominantly elderly, the range of eGFR values was relatively small and albuminuria was present only in a small minority. These observations are nevertheless important because our study cohort is representative of the majority of people affected by early stage CKD, at least in the UK.

Previous studies have also identified age, blood pressure and the presence of diabetes as determinants of higher aPWV [6], [17], [20]. Our observation that aPWV increased to a greater extent with age in males versus females is consistent with data from another study that identified male gender as an independent determinant of increased aPWV in a large cohort of people with CKD [17].The increase in AS with age is proposed to be due to overproduction of abnormal collagen fibres and a loss of elastin from the extracellular matrix [9], [37]. It is not clear, however, whether this is a time dependant phenomenon directly related to chronological age or if it reflects exposure to other risk factors. Hypertension has long been recognised as a major determinant of arterial stiffness due to the associated medial hypertrophy [38]. The association between diabetes and arterial stiffness may be due to accumulation of advanced glycation endproducts (AGE) that provoke structural changes in the arterial wall [22] and the generation of reactive oxygen species that deactivate nitric oxide resulting in endothelial dysfunction [39].

BMI had an inverse relationship with aPWV. This is surprising because AS has previously been associated with obesity, particularly abdominal obesity [40], and increased waist to hip ratio was associated with higher aPWV in our univariate analysis. We have previously described that BMI decreased with age in our cohort, likely reflecting a loss of muscle mass [21]. Our observation may therefore be explained by lower BMI acting as marker of increased age (the dominant determinant of aPWV) that could not be completely corrected for in the multi-variable analysis. Furthermore, we have previously shown that measures of obesity that include central fat distribution are more closely related to important risk factors in those with CKD than BMI [41].

There are a several limitations to this study. First, this analysis includes only cross-sectional data and we are therefore unable to draw any firm conclusions regarding possible causal relationships between AS and the determinants identified. However, the planned 10-year follow up of the cohort will allow us to investigate the factors that contribute to AS prospectively. Many studies have used aplanation tonometry devices (SphygmoCor™ or Complior™) to measure PWV [17], [19], [20], but these are operator dependant, time consuming to use and not easy to transport. We therefore used the more portable Vicorder™ device which is also operator independent and requires only minutes to obtain a reading. Vicorder™ measurements of aPWV have been shown to be reproducible and correlate well with SphygmoCor™ measurements [42] but as yet there is no agreed method for making direct comparisons between results obtained by different methods [43]. A third limitation of our study is the absence of normal controls. Finally, participants comprised only 22% of people invited to participate and may therefore not be representative of all people with CKD stage 3. We were unable for ethical reasons to obtain data on non-participants but our study population was similar to a large population of people with CKD stage 3 derived by pooling data on all patients from several GP databases, suggesting that our participants were broadly representative of patients with CKD stage 3 in primary care in the UK, though with a low proportion of people from ethnic minorities [44]. Strengths of the study include the large cohort size, robust measures of eGFR and UACR, and detailed clinical assessment of each participant. A single operator performed all assessments, eliminating inter-observer variability.

### Conclusion

Age was the strongest determinant of arterial stiffness in this cohort of predominantly elderly patients with CKD stage 3. Other significant independent determinants of aPWV included traditional cardiovascular risk factors such as blood pressure, diabetes, obesity and a marker of dyslipidaemia. Markers of CKD were associated with aPWV only in univariate analysis (eGFR) or were weak determinants of aPWV (albuminuria). Long term follow-up will investigate the importance of arterial stiffness as an independent risk factor for cardiovascular events in this cohort.
